# 小细胞肺癌分子分型：向临床实践转化的挑战

**DOI:** 10.3779/j.issn.1009-3419.2024.106.21

**Published:** 2024-08-20

**Authors:** Zhihong LIN, Lei FAN, Ping HE

**Affiliations:** 510120 广州，广州医科大学附属第一医院病理科; Department of Pathology, First Affiliated Hospital of Guangzhou Medical University, Guangzhou 510120, China

**Keywords:** 肺肿瘤, 小细胞肺癌, 分子分型, Lung neoplasms, Small cell lung cancer, Molecular subtype

## Abstract

小细胞肺癌（small cell lung cancer, SCLC）是肺癌的组织学亚型之一，特点是高增殖、早转移、易耐药和复发。数年来SCLC一直被视为一种同质性疾病，治疗采用统一的放化疗策略。尽管早期疗效显著，但耐药和复发很快出现，缺乏满意的治疗效果，这可能是因为目前对SCLC的肿瘤异质性认识不足所导致。最近，临床前研究提出了基于谱系转录因子相对高表达定义的SCLC分子分型概念。本文主要阐述SCLC分子分型的现状和相关最新发现，强调分子分型转化在临床实践中所遇到的问题，旨在增进对SCLC分子分型研究进展的认识。

## Abstract (en)

Small cell lung cancer (SCLC), one of the histological subtypes of lung cancer, is characterized by high proliferation, early metastasis, susceptibility to drug resistance and recurrence. For several years, SCLC has always been regarded as a homogeneous disease, treated with a unified radiotherapy and chemotherapy strategy. Despite significant early therapeutic effects, drug resistance and recurrence occur quickly, and there is a lack of satisfactory treatment results, which may be due to insufficient understanding of the tumor heterogeneity of SCLC at present. Recently, the concept of SCLC molecular subtype based on the definition of relatively high expression of lineage transcription factors has been proposed in preclinical studies. This article mainly elaborates on the current status and latest findings of SCLC molecular subtype, emphasizing the potential problems that molecular typing may encounter in clinical practice, aiming to promote understanding of the research progress of molecular subtype in SCLC.

肺癌是导致人类癌症相关死亡的主要原因。根据全国肿瘤登记中心的数据^[1]^，估计2022年中国恶性肿瘤新发病例数为482.47万，其中肺癌居恶性肿瘤发病首位，2022年肺癌新发病例数约106.06万，约占全部恶性肿瘤的22.0%。北美癌症登记机构（North American Association of Central Cancer Registries）数据^[2]^显示，仅2023年美国估计新增肺癌病例达238,340例，估计新增肺癌相关死亡病例达127,070例。小细胞肺癌（small cell lung cancer, SCLC）约占所有肺癌的15%，5年生存率通常低于7%。针对异常基因靶向用药的策略已在非小细胞肺癌（non-small cell lung cancer, NSCLC）治疗中取得巨大进展，相比之下，SCLC仍未建立个体化治疗的蓝图。当前的治疗指南中，依托泊苷+铂类药物的化疗组合仍作为SCLC一线标准治疗的基础。研究^[3,4]^证实了免疫治疗在广泛期小细胞肺癌（extensive stage SCLC, ES-SCLC）的临床效益，结果显示，与传统化疗相比，化疗+免疫治疗的治疗策略延长了ES-SCLC患者的总生存期（overall survival, OS）和无进展生存期（progression-free survival, PFS）。基于两项大型临床试验CASPIAN^[3]^和IMpower133^[4]^的数据结果，免疫治疗药物阿替利珠单抗（Atezolizumab）和度伐利尤单抗（Durvalumab）两种程序性死亡配体1（programmed cell death ligand 1, PD-L1）抑制剂被美国食品药品监督管理局（Food and Drug Administration, FDA）批准用于ES-SCLC一线治疗。我国自主研发的PD-1抑制剂“斯鲁利单抗”在ES-SCLC患者生存获益中亦有较好的表现，并在2023年纳入中国临床肿瘤学会（Chinese Society of Clinical Oncology, CSCO）指南ES-SCLC的一线治疗（证据类别：1A类）^[5]^。尽管免疫疗法使SCLC相对单一的治疗手段得到了补充，然而临床研究数据^[6,7]^显示，对免疫检查点抑制剂（immune checkpoint inhibitors, ICIs）有反应活性的SCLC仅约占15%，SCLC患者整体从免疫治疗中获益有限。因此，迫切需要对SCLC分子特征进行探索，深入认识SCLC的肿瘤异质性，以发掘更多的分子靶点。

## 1 SCLC分子病理学特征

SCLC发生的分子机制目前尚未完全阐明，研究^[8,9]^表明吸烟与SCLC发病风险密切相关，与NSCLC相比，SCLC有着不同的分子途径。全基因组学分析揭示了SCLC一些关键的突变，约90%的SCLC具有肿瘤抑制基因TP53和RB1功能性失活，并且证实了抑癌基因的双失活性突变在SCLC发生中的关键作用^[10,11]^，其他较为常见的突变基因包括：性别决定区Y框蛋白2（SRY-box transcription factor 2, SOX2）、环腺苷酸反应元件结合蛋白（CREB binding protein, CREBBP）、Notch1受体（Notch receptor 1, NOTCH1）、腺病毒E1A结合蛋白P300（E1A binding protein P300, EP300）、肿瘤蛋白P73（tumor protein P73, TP73）、Slit同源物2（Slit guidance ligand 2, SLIT2）、张力蛋白同源物（phosphatase and tensin homolog, PTEN）、成纤维细胞生长因子受体1（fibroblast growth factor receptor 1, FGFR1）及MYC（Myelocytomatosis）家族基因等^[10,12⇓-14]^。最近，有证据^[15]^表明SCLC可基于几个关键转录因子即神经母细胞特异性转录因子1（achaete-scute homologue 1, ASCL1）、神经源分化因子1（neurogenic differentiation factor 1, NEUROD1）、POU2类同源框3（POU class 2 homeobox 3, POU2F3）和Yes相关蛋白1（Yes-associated protein 1, YAP1）的相对表达水平区分为几种亚型，分别命名为SCLC-A、SCLC-N、SCLC-P、SCLC-I和SCLC-Y，ASCL1-/NEUROD1-/POU2F3-三者低表达构建为SCLC-I（inflamed）。值得注意的是，YAP1在SCLC中表达量较低且在免疫组化中常未检测到其高表达，故部分观点认为SCLC-Y不能够构建为独立的亚型，图1展示SCLC不同分子亚型及其高表达的靶分子。

**图1:**
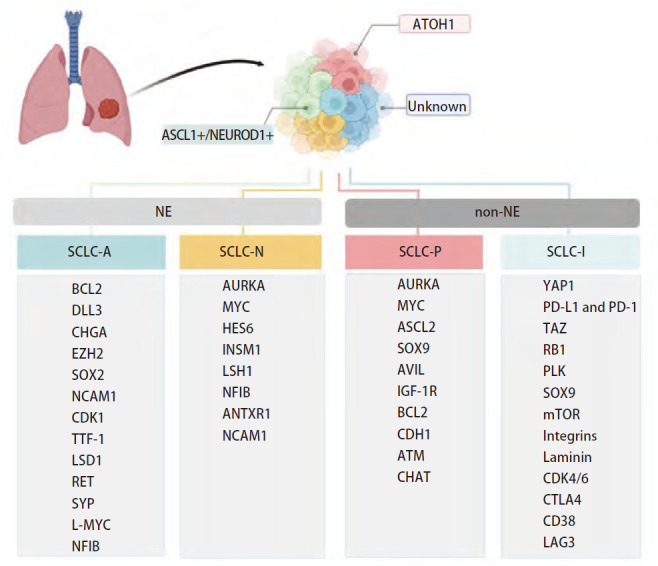
SCLC肿瘤分子分型及其靶分子

## 2 SCLC分子分型的主要变化

SCLC的分类经历了多个阶段的发展，早期对于SCLC分类的探索在1985年，Carney等^[14]^发现人类SCLC细胞系可根据肿瘤细胞生物学行为和神经内分泌（neuroendocrine, NE）表型区分为NE干细胞样的经典型（classic）和非NE（non-NE）干细胞样的变异型（variant）两大类。

2019年，Rudin等^[15]^根据SCLC细胞系、人类SCLC肿瘤样本及患者来源异种移植物（patient-derived xenograft, PDX）的测序数据，提出一种广为接受的SCLC分型概念，基于4个关键转录调控因子（ASCL1、NeuroD1、YAP1及POU2F3）的相对表达量，将SCLC分为4种肿瘤亚型，并分别命名为SCLC-A、SCLC-N、SCLC-Y和SCLC-P。SCLC-A和SCLC-N为NE表型，ASCL1和NEUROD1在促进细胞正常NE表型的形成和部分SCLC的发生中起着重要作用，它们分别能结合不同基因位点，从而调节大多数独特的致癌基因，如ASCL1能靶向的致癌基因包括MYCL原癌基因BHLH转录因子（MYCL proto-oncogene BHLH transcription factor, MYCL1）、重排致癌基因（rearranged during transfection, RET）、SOX2和核因子I/B（nuclear factor I/B, NFIB）等，还能调节NOTCH信号通路中的多个基因，包括德尔塔样蛋白3（delta-like canonical Notch ligand 3, DLL3）；NEUROD1则能靶向MYC^[16]^，SCLC-P和SCLC-Y为non-NE表型，转录调控因子YAP1和POU2F3是non-NE SCLC细胞生长所必需的因子。研究^[17]^表明NE SCLC相比non-NE SCLC更具侵袭性。

随后Wooten等^[18]^发现高表达ASCL1的SCLC亚型可进一步细分为SCLC-A和SCLC-A2两个子型，其中SCLC-A2子型更具有耐药性。此外，Simpson等^[19]^在患者循环肿瘤细胞来源模型（circulating-tumor-cell derived explant models）中识别出一个先前未报道的、高表达ATOH1的SCLC亚型，但该亚型的存在还需要在更多样本中得到验证。Baine等^[20]^研究发现相对于ASCL1、NEUROD1和POU2F3的表达水平，YAP1表达水平相当低。Chan等^[21]^测序分析了21例新鲜SCLC临床样本中155,098个细胞的转录组数据未能识别出SCLC-Y。Baine等^[20]^和Megyesfalvi等^[22]^分别通过对原发肿瘤和循环肿瘤细胞移植物利用免疫组化方法均未能识别YAP1亚型。这与SCLC中免疫组化通常观察到YAP1的低表达现象一致，表明需要更多的研究证据支持YAP1能否代表独立的亚型。后续的研究中，Owonikoko等^[23]^通过免疫组化方法成功地将99例SCLC中的绝大部分病例区分为SCLC-A、SCLC-N、SCLC-P和SCLC-Y四种亚型中的一种，但仍有32%的病例不属于以上的任何一种。

2021年Gay等^[24]^对81例手术切除SCLC样本的RNA测序数据利用非负矩阵因子分解方法识别并区分为四种SCLC亚型（SCLC-A、SCLC-N、SCLC-P、SCLC-I），SCLC-A、SCLC-N和SCLC-P如前所述，SCLC-I为三个转录因子（ASCL1、NEUROD1和POU2F3）均低表达但独特表达许多免疫检查点和人类白细胞抗原（human leukocyte antigens, HLAs）相关基因，SCLC-I能从化疗联合免疫治疗的组合中有较高的获益，Gay的研究建议用SCLC-I取代YAP1亚型（SCLC-Y）。

2022年Qu等^[25]^在146例原发SCLC中通过免疫组化方法观察到ANPY四阴性（ANPY-）即SCLC-QN，这可能识别为一种新的SCLC亚型。目前，SCLC分子分型仍在探索与变化中（图2）。

**图2:**
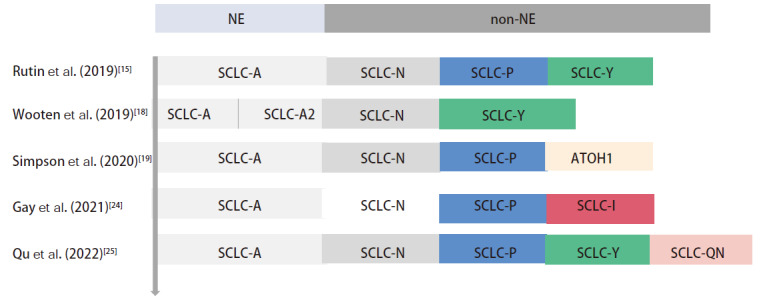
基于差异基因表达定义SCLC亚型的主要研究

## 3 SCLC分子分型的临床前治疗

不同SCLC亚型反映了肿瘤细胞的NE分化程度、细胞代谢的差异以及不同基因的差异表达，更重要的是SCLC亚型具有各自的治疗敏感性并且针对治疗靶点的药物已在研究中。

### 3.1 SCLC-A

可作用靶点包括BCL2和DLL3^[26]^。在SCLC-A中观察到BCL2上调，提示BCL2可能是ASCL1的下游靶点^[16]^。研究^[27]^显示BCL2抑制剂对SCLC的抑制作用与BCL2的表达相关，因此，BCL2抑制剂拟作为针对SCLC-A的靶向药在研究中。Notch信号通路在细胞中发挥抑癌作用，DLL3是Notch通路的配体，由ASCL1调节，DLL3与DLL1通过与Notch通路受体相互作用抑制Notch通路的激活从而促进SCLC发生^[16,28,29]^。因此靶向DLL3可作为治疗SCLC的手段之一，相关的临床前研究药物包括抗体-药物偶联物、双特异性T细胞接合剂（bispecific T cell engagers, BiTE）。嵌合抗原受体T细胞（chimeric antigen receptor T, CAR-T）构建体是一种靶向DLL3的抗体-药物偶联物，名为罗伐尔匹珠单抗-替西林（Rovalpituzumab Tesirine, Rova-T），由一种人源化抗DLL3抗体组成，该抗体与DNA损伤毒素吡咯并二氮唑平的二聚体偶联^[30]^，然而针对DLL3靶点的试验药物Rova-T在III期临床试验（TAHOE）中未能突显令人满意的结果^[31]^，尽管如此，DLL3作为治疗靶点的其他几项临床试验^[32,33]^仍在进行中。

### 3.2 SCLC-N

MYC是NEUROD1的下游靶点，其表达与SCLC的多种治疗敏感性相关。Owonikoko等^[34]^发现AURK激酶抑制剂对MYC高表达的SCLC有效，在复发性SCLC患者中联合使用紫杉醇（Paclitaxel, PTX）和阿利赛替布（Alisertib, ALS），结果显示联合用药使患者PFS增加了1倍，但仅在MYC阳性肿瘤患者中观察到。Chalishazar等^[35]^研究发现MYC驱动的SCLC亚型有代谢异质性，用聚乙二醇化精氨酸脱氨酶（arginine deiminase-polyethylene glycol 20, ADI-PEG20）耗尽精氨酸可显著抑制其生长，并在小鼠、细胞系和PDX模型上得到验证，提示精氨酸耗竭有可能作为MYC驱动的SCLC 的特异性治疗手段。

### 3.3 SCLC-P

相比其他亚型，SCLC-P对PARP（poly ADP-ribose polymerase）抑制剂更为敏感。其他特异性治疗手段包括针对靶分子SRY盒9转录因子（SRY-box transcription, SOX9）和ASCL2（achaete-scute family BHLH transcription factor 2）以及胰岛素样生长因子1受体（insulin-like growth factor 1 receptor, IGF1R）的抑制剂^[36]^。最近，POU2F3被认为可用作NE标志物低表达或缺失表达SCLC的一种实用有效的诊断标志物^[37]^，在Wang等^[38]^的大型研究队列中，其敏感性和特异性分别为82.1%和99.4%。

### 3.4 SCLC-Y

SCLC-Y是non-NE、炎症相关表型（inflamed），与干扰素γ反应基因、T细胞受体基因、HLA基因、抗原呈递机制基因和T细胞炎症基因的高表达水平有关。SCLC-Y的潜在治疗手段包括ICIs、哺乳动物雷帕霉素靶蛋白（mammalian target of rapamycin, mTOR）抑制剂、Polo样激酶1（Polo-like kinase 1, PLK1）抑制剂、YAP1抑制剂。

### 3.5 SCLC-I

SCLC-I具有许多免疫检查点和HLAs相关基因高表达，也有较多的免疫相关淋巴细胞的浸润，预示其在ICIs联合治疗中能有更多的获益。III期临床试验IMpower133（NCT02763579）^[4]^评估了在一线标准化疗方案EP中联合加入阿替利珠单抗（Atezolizumab）对ES-SCLC患者的疗效，结果显示与单独接受EP[依托泊苷（Etoposide）+顺铂（Cisplatin）]方案治疗的患者相比，EP方案联合阿替利珠单抗（Atezolizumab）治疗SCLC‐I患者的中位生存期（median overall survival, mOS）高出约8个月，OS延长2个月，死亡风险降低30%。研究^[24]^表明SCLC-I细胞有布鲁顿酪氨酸激酶（Bruton tyrosine kinase, BTK）的高表达，使其对BTK抑制剂（如伊布替尼）具有潜在治疗敏感性。

## 4 SCLC分子分型向临床实践转化中的问题和挑战

SCLC分型方法和治疗手段最终转化为临床实践的前提是科学性，尽管分子分型显示出良好的临床前景，我们仍需要了解推动其未来应用于临床目前尚待解决的问题。

### 4.1 研究样本的不足

由于SCLC发展极为迅速和早期转移，约2/3患者确诊时已出现远处转移，仅肿瘤原发灶-淋巴结-转移（tumor-node-metastasis, TNM）I、II期（T1-2, N0, M0）或部分局限期SCLC患者适宜接受手术，实际上进行手术切除的患者仅占极小部分，这使得能够用于研究的手术标本十分有限并且标本大多为早期SCLC。基因工程小鼠模型（genetically engineered mouse models, GEMMs）、PDX和SCLC细胞系的创建填补了研究样本上的不足，值得注意的是，这些临床前模型实际上相当于SCLC发展的早期阶段^[39]^。最近，利用CRISPR/Cas9介导的体细胞基因编辑的方法进行SCLC小鼠建模具有方便、快捷和低成本的优势，有希望加速针对SCLC致癌候选基因的功能性研究^[40]^。同时，SCLC具有丰富的循环肿瘤细胞（circulating tumor cells, CTCs）浓度，通过液体活检获取SCLC细胞及循环肿瘤DNA（circulating tumor DNA, ctDNA）检测在诊断及监测疾病方面具有潜在作用^[41]^。

### 4.2 肿瘤异质性

根据预先定义的谱系标志物和判读方法，多数SCLC能被区分为不同的亚型，然而在一部分研究^[42,43]^中观察到了SCLC存在的肿瘤异质性，组织学上表现为：（1）病理切片中观察到表达ASCL1的肿瘤细胞与表达NEUROD1的肿瘤细胞在不同区域共存；（2）存在两种或多种标志物共表达的肿瘤细胞。肿瘤的异质性表达将使得分子分型在临床实践判读中存在一定的分类困难。肿瘤细胞亚群间可能并不是完全互斥的，它们的相对表达是动态灵活的，可随着肿瘤的进展发生变化，仅通过单一标志物相对量来定义亚型有可能在一定程度掩盖了其他肿瘤细胞亚群的特性从而低估了肿瘤异质性。SCLC分子分型的结果能否真实反映实体肿瘤中不同肿瘤细胞亚群的分类和占比尚需要多中心大样本数量的研究证据支持。

### 4.3 分子分型的可重复性和临床适用性

Baine等^[20]^用免疫组化方法将174例SCLC患者样本按ASCL1、NEUROD1、POU2F3和YAP1四种转录因子的相对表达分类，得出结果SCLC-A/-N/-P分别占比69%、17%、7%，YAP1表达水平较低并且主要存在于复合型SCLC中，其未能代表一个独立的亚型，仍有7%的SCLC未能归属于任何一个亚型。Owonikoko等^[23]^利用shinyISPA技术（http://bbisr.shinyapps.winship.emory.edu/shinySISPA）根据ASCL1、NeuroD1、YAP1和POU2F3基因的表达谱对59例NE肿瘤（其中33例SCLC）进行分类，结果显示有14例SCLC未能属于四种亚型中的任何一种。同样地，Qu等^[25]^利用免疫组化对146例SCLC患者原发肿瘤样本分类，有6.3%的病例对上述A、N、P、Y四种亚型标志物均呈阴性而未能归为任何一个亚型。由此可见，不同研究的分类结果不完全一致，这可能因为研究分子水平的不同或是技术方法不同，免疫组化检测也可能因组织样本来源、检测平台、抗体试剂克隆号和H-score阳性截值等因素不同而导致结果相差较大。刘丽^[44]^通过对比SCLC在mRNA和蛋白质层面分类的结果认为SCLC的NE分型在mRNA和蛋白质水平间有显著一致性，分子分型在mRNA和蛋白质水平间的结果一致性较差。Szeitz等^[41]^的研究结果显示蛋白质表达检测的SCLC分型与基于mRNA的分型一致，但认为基于转录谱的分型可能并不完全代表基于蛋白质表达的分型。

### 4.4 细胞起源

肺上皮包含多种细胞类型，由于SCLC的NE特征，SCLC通常被认为起源于肺内的NE细胞，同时独立研究也证实了NE细胞在TP53和RB1双失活的条件下可发展为SCLC。尽管一些研究表明SCLC也可起源于2型肺泡上皮细胞（alveolar epithelial type II cell, AT2），但与NE细胞相比，这些细胞的SCLC转化效率和转化率较低^[45⇓-47]^ 。SCLC-P表现出与簇状细胞（tuft cell）相同的基因表达，因此也被指定为簇状细胞样SCLC，提示SCLC-P可能与其他亚型有着不同的细胞起源。然而与NSCLC不同的是，组织学上SCLC未能观察到从癌前病变到浸润性癌的过程，这使得对于SCLC的细胞起源仍然缺乏直观的认识。目前尚未知SCLC不同的亚型是否代表了不同的细胞起源，需要更多的研究证据证实这是否反映了SCLC不同的起源细胞或分化。

### 4.5 分子亚型间的转变

尽管SCLC目前已被识别出根据不同转录因子高表达的亚型，这些亚型之间的关系尚未完全清楚。Ireland等^[48]^在小鼠和人类肿瘤样本中验证了MYC激活Notch信号通路使NE型SCLC去分化从ASCL1+到NEUROD1+再到YAP1+的non-NE型的动态演变，并且这种动态演变可发生在患者肿瘤中（图3），提示亚型的结果有可能是SCLC进行性进化的不同阶段，即分子分型的结果可能是SCLC动态性演变的静态呈现。ASCL1+/NEUROD1+共表达的SCLC细胞有可能为一种过渡状态。Chiang等^[43]^的研究中对12例SCLC患者疾病进展后重新活检，发现6例（50%）患者发生了SCLC亚型转变，具体而言，3例从SCLC-A转为SCLC-N，1例从SCLC-A转为SCLC-NAPY-（四阴性），2例从SCLC-NAPY-（四阴性）转为SCLC-N。SCLC不同亚型之间是具有一定的进化轨迹还是遗传学等因素作用之下的静态呈现尚未知，需要进一步阐明这些不同亚型之间的关系。SCLC分子亚型可能只是阶段性的，并不能代表其所有特征，如何更好地表征其分子异质性对于准确识别肿瘤的分子状态，尤其在肿瘤发生转化时进行有效的识别和干预是至关重要的：（1）多区域活检和多时间点采样能够更全面地捕捉肿瘤的空间和时间异质性，有利于临床医生及时发现亚型的转变从而更改治疗方案；（2）单细胞RNA测序（single-cell RNA sequencing, scRNA-seq）可以识别出肿瘤中不同分子亚型的混合，以及它们在治疗过程中的变化；（3）开发生物标志物，识别并验证SCLC异质性相关的生物标志物，有助于在临床上监测肿瘤状态的变化，尤其是与特定亚型或转化相关的标志物，有助于排除因异质性引起的假阳性结果，并更准确地判断是否存在亚型的转化；（4）期望整合基因组学、转录组学、蛋白质组学和表观基因组学的多组学分析提供更多的数据以建立多维度的分子分型框架。Gay等^[24]^的研究指出亚型的转变可能是SCLC获得耐药性的机制，提示未来针对单一亚型治疗的成功关键可能需要通过靶向表观遗传学调节因子来实现阻断亚型之间的转变或重编程进而针对性的治疗。

**图3:**
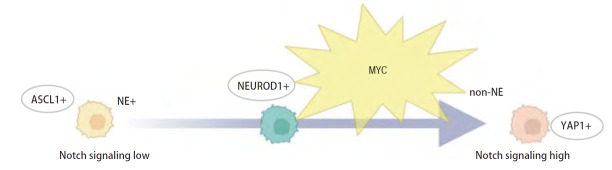
MYC通过重新编程神经内分泌特性驱动SCLC亚型随时间转变

### 4.6 肿瘤原发灶与转移性淋巴结间谱系标志物的表达差异

多数SCLC患者中，淋巴结转移灶等小活检往往是疾病确诊的最初证据。一个未来待解决的问题是通过淋巴结等小活检评估分子分型和治疗相关标志物的表达是否能够代表原发性肿瘤的表达?这对于临床医生选择何种术后辅助治疗和评估治疗效果至关重要。目前，关于SCLC肿瘤原发灶与淋巴结转移灶间分子分型标志物表达模式的研究较少且不一致，Handa等^[42]^的研究结果表明肿瘤相关巨噬细胞和淋巴结转移灶的谱系标志物阳性率与相应手术标本的阳性率显著相关，认为活检标本可用于识别患者SCLC的分子亚型，而最近的一项研究^[49]^结果认为SCLC原发性肿瘤和淋巴结转移灶之间分型谱系标志物和治疗标志物表达不具有一致性。

## 5 小结与展望

目前，SCLC仍缺乏经批准的分子靶向药物和指导用药的分子标志物，分子分型实际临床应用价值尚不充足，期待未来更新的多组学研究证据结合分子和免疫学特征进一步深入和细化SCLC分类并转化为临床实践，以期进行更精确地诊断、预测预后和制定个体化治疗方案。
